# Mutation analysis of *Netrin 1* and *HMX3* genes in patients with superior semicircular canal dehiscence syndrome

**DOI:** 10.3109/00016489.2012.681797

**Published:** 2012-07-10

**Authors:** Nikola Roknic, Alexander Huber, Stefan C. A. Hegemann, Rudolf Häusler, Nicolas Gürtler

**Affiliations:** ^1^Klinik für Hals-Nasen-Ohrenkrankheiten, Hals- und Gesichtschirurgie, Kantonsspital Aarau AG; ^2^Klinik für Ohren-, Nasen-, Hals- und Gesichtschirurgie, Universitätsspital Zürich; ^3^Universitätsklinik für Hals-, Nasen-, Ohrenkrankheiten, Kopf- und Halschirurgie, Inselspital Bern; ^4^Hals-Nasen-Ohren-Universitätsklinik, Universitätsspital Basel, Switzerland

**Keywords:** Genetics, vestibular development

## Abstract

**Conclusion:**

In spite of its absence in the control population, there is questionable evidence for the alteration c.114C->T in the *HMX3* gene being implicated in the development of superior semicircular canal dehiscence (SSCD). However, the concept of a complex disease is valid for SSCD and a possible molecular origin can neither be confirmed nor excluded by the results of this study.

**Objectives:**

SSCD was first described in 1998 by Minor et al. While the etiology is not clear, findings from both temporal bone CT and histologic studies suggest a congenital or developmental origin. In recent years, a couple of genes regulating inner ear morphogenesis have been described. Specifically, *Netrin-1* and *HMX3* have been shown to be critically involved in the formation of the SCC. Molecular alterations in these two genes might lead to a disturbed development of this canal and might represent an explanation for SSCD.

**Methods:**

DNA was extracted from whole blood of 15 patients with SSCD. The coding sequences of *Netrin-1* and *HMX3* were amplified by PCR and sequenced.

**Results:**

One sequence alteration, heterozygous c.114C->T (conservative change without alteration of amino acid) in exon 1 of *HMX3,* was detected in 2 of 15 patients but not in 300 control chromosomes. The study was supported in part by the Emilia-Guggenheim-Schnurr-Foundation, Basel, Switzerland.

## Introduction

The syndrome of superior semicircular canal dehiscence (SSCD) was identified by Minor et al. in 1998 [1]. Typically patients complain about vertigo and oscillopsia, which are induced by loud sounds or changes in middle ear pressure such as the Valsalva maneuver. Clinically, eye movements can be elicited by loud noises (Tullio phenomenon) or by pressure in the external auditory canal (Hennebert sign). As bone conduction thresholds can be less than 0 dB normal hearing level, an air conduction threshold above 0 dB can create a ‘false' air–bone gap. Rinne tuning fork testing is positive and the acoustic stapedius reflexes are present.

The dehiscence of a variable extent of the bone overlying the SCC has been demonstrated by high-resolution computed tomography (HRCT) of the temporal bones in axial and coronal planes with 0.5 mm collimation with a sensitivity of 100% and a specificity of 99% [2,3]. Additional confirmation of the dehiscence stems from intraoperative findings and from a temporal bone study [3,4]. The etiology remains unknown. However, findings from both the CT and histological study point to a congenital or developmental origin [2,4]. With this in mind, a molecular-genetic defect might be the cause for SSCD. The fact that familiality has so far not been reported for SSCD does not exclude such an approach. Indeed, an incomplete penetrance of this syndrome with 2% asymptomatic individuals due to variable bone thickness at the middle fossa floor was observed in the temporal bone study [4]. The identification and characterization of genes controlling the development of the vestibular organs have advanced in recent years. More specifically, two genes, *HMX3* (Nkx5-1) and *Netrin-1*, which are critically involved in the formation of the SCC, have been published [5,6]. Both genes are expressed in the non-sensory vestibular epithelium. Mutant mice show lack or reduction of any of the three semicircular canals, predominantly the superior one, and do not display any morphologic or histologic abnormality of the cochlea. While *HMX3* is suggested to act as a transcriptional repressor, *Netrin-1* is considered to represent a secreted protein, related to laminin, and might be involved in morphogenesis and cell migration. In addition, periotic mesenchyme is diminished in *Netrin-1* mutant mice, indicating a role for *Netrin-1* for inducing bony duct formation. Additional evidence for *Netrin-1* activity in tissue morphogenesis stems from the report of its role in the development of the mammary gland terminal end buds [7].

The aim of this study was to analyze these two genes for a possible genetic cause or predisposition in patients with SSCD.

## Material and methods

### Patients

Patients with SSCD were recruited for the study. The diagnosis of SSCD was based on the results of the thin-sliced CT of the temporal bone. History and neurotologic and auditory findings were collected. Informed consent, according to the guidelines approved by the Ethics Committee of the University Hospital of Basel, was obtained from each individual participating in the study.

### Molecular analysis

Genomic DNA was extracted from whole blood using the Qiagen DNA isolation kit (QIAamp DNA BloodMaxi Kit®, Basel, Switzerland). Coding regions and exon–intron boundaries of the *Netrin-1* and *HMX3* genes (GenBank accession nos NM_004822.2 and NM_001105574.1) were investigated by sequencing analysis. The single coding exon of the two genes was amplified in overlapping fragments using the primers listed in the Appendix.

Three types of master mixes were used for amplification: True Allele Mastermix (Applied Biosystems, Rotkreuz, Switzerland) for NT2b and NT4; True Allele-DMSO Mastermix (Applied Biosystems) for NT2a, NT2c, NT3, NT5, HMX 3.2a, and HMX 3.2b; and the GC-Rich-PCR System (Roche Diagnostics, Rotkreuz, Switzerland) for NT7 and HMX3.1. The fragments were amplified by the following PCR programs: 94°C for 10 min; 35 cycles of 94°C for 15 s, 60°C for 20 s, 72°C for 40 s, and final extension of 72° for 5 min; and for exons NT7 and HMX3.1, 97°C for 5 min, 35 cycles of 97°C for 30 s, 58°C for 30 s, 72°C for 30 s, and final extension at 72°C for 7 min. After PCR amplification, excess primers and dNTP were removed by Exosap-IT® (Amersham Biosciences, Otelfingen, Switzerland) according to the manufacturer's protocol. The sequencing reaction was performed using the Big Dye Terminator Cycle Sequencing kit (Applied Biosystems) according to the manufacturer's guidelines. After purification using the DyeEx 2.0 Spin Kit (Qiagen, Basel, Switzerland) sequencing products were run on an ABI PRISM 310 Genetic Analyzer (Applied Biosystems). The sequences were analyzed using the Sequencher 4.0 software (Gene Codes Corporation, Ann Arbor, MI, USA). Each fragment was sequenced twice following independent PCR amplification to ensure accurate sequence results.

## Results

### Patient characteristics

Fifteen patients with SSCD were included in the study, six women and nine men. The median age was 50 years with a range of 38–67 years. All patients had evidence of superior canal dehiscence on HRCT and were of caucasian origin. Three (20%) of them had a dehiscence on both sides, four (26%) had right-sided SSCD, and eight (54%) patients had left-sided SSCD. Seven of them underwent surgical treatment by plugging or resurfacing the SSC. A wide range of symptoms was noted (Table I). Auditory manifestations were identified in seven (46%) patients and included symptoms such as autophony, pulsatile tinnitus, hyperacusis or hearing of cervical spine movements. Eight patients (55%) complained about vestibular symptoms such as the Tullio phenomenon, dizziness or vertigo. An air–bone gap was measured in 10 patients (67%).

**Table I.** Patients' symptoms and clinical findings.

Characteristic	No. of patients (%)
Symptoms	
Pressure-induced	5 (33)
Induced by loud sounds	7 (47)
Clinical findings	
Air–bone gap >10 dB HL	10 (67)
Lower threshold in vestibular evoked myogenic potentials	7 (47)

### Molecular analysis

**Figure 1.**
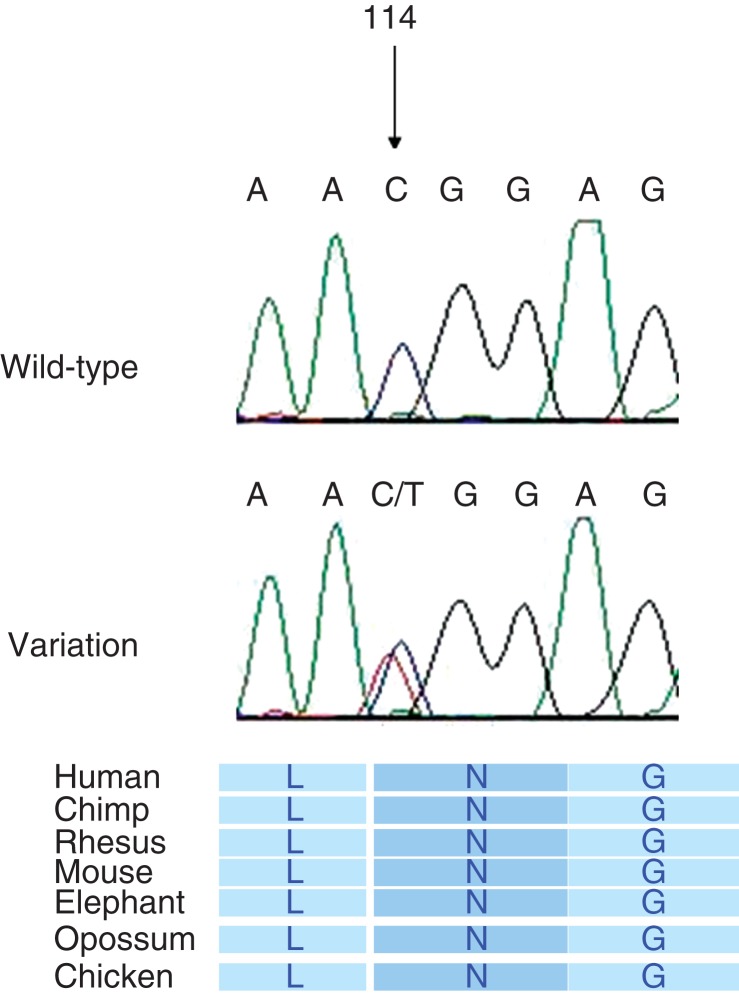
Novel polymorphism heterozygous c.114C->T (p.N114N) in exon 1 of *HMX3*, showing the conservative nature across species.

Mutation analysis of *Netrin-1* and *HMX3* revealed one single sequence alteration in the coding sequences and intron–exon junctions of these genes after comparison with the published cDNA sequences. The alteration c.114C->T was found in heterozygous form in exon 1 of gene *HMX3* in two patients and was classified as conservative as the amino acid asparagine was not changed: p.N38N (Figure 1). C.114C->T was not found in any genomic database and therefore represents a novel polymorphism. However, it was not detected in 300 control chromosomes of caucasian ethnicity.

## Discussion

SSCD is a rare disease, whose underlying pathophysiology remains unknown. Radiologic and histologic evidence points to a developmental cause, which presumably occurs postnatally [2,4]. The morphogenesis of the vestibular organ is a highly complex procedure. The semicircular ducts emerge as protuberances of the dorsal vestibular epithelium. These protuberances come together to form the so-called fusion plate, which acts as a precursor of the duct epithelium. Close interaction with the periotic mesenchyme allows proper chondrification of the semicircular ducts [6]. Although a number of genes have been reported to be implicated in the formation of the semicircular canals based on animal studies, the number of the various involved genes, the exact mechanism and their interactions are still largely unknown [8]. However, two genes in particular, *Netrin-1* and *HMX3*, have been shown to be critically involved in semicircular duct formation [5,6]. *HMX3* and *Netrin-1* mutant mice show missing posterior and lateral semicircular canals, normal sensory epithelium, and an intact cochlea.

This study analyzed the coding sequences of the *Netrin-1* and *HMX3* genes in 15 patients with SSCD. After sequencing analysis of the coding regions and exon–intron boundaries, one single variation, c.114C->T, in *HMX3* was found in two patients (13%). The conservative change and its low frequency in the patients suggest primarily a nonfunctional role in spite of its lack of identification in the normal population. Bioinformatic analysis revealed a possible functional role, as the nucleotide change at position 114 from C to T in the *HMX3* gene abolishes a binding site for the RS-protein SF2/ASF according to ESEfinder 2.0 software (http://rulai.cshl.edu/tools/ESE2/). However, the question as to whether or not this reduction by one SF2/ASF binding site (there are several SF2/ASF binding sites remaining) alone influences the splicing activity in the native *HMX3* pre-mRNA remains to be answered.

Recently, four children with hemizygous deletions on chromosome 10, harboring the *HMX3* gene, have been reported [9]. Besides numerous anomalies, such as dysmorphic features, all patients exhibited delayed and unsteady walking. On CT scan the ratio of the transverse dimension of membranous vestibule to the inner diameter of the lateral SCC and an abnormally enlarged cystic vestibule were detected. None of our adult patients showed features compatible with this report.

No additional sequence alterations were found in any of the patients. One explanation might be the fact that we cannot exclude either mutations in the *cis* transcription regulatory elements or promoter region, or possible cryptic splice-site mutations. Splice-site mutations in the inner ear, for example, have been described for *Pendrin*, a deafness gene causing Pendred syndrome and enlarged vestibular aqueduct [10]. In addition, homozygous deletions can be ruled out, as all exons of the two genes could be amplified in all patients. Complex inheritance could be another explanation for the development of SSCD. Complex or multifactorial origin has been reported with increasing evidence for otosclerosis or age-related hearing loss [11]. The low frequency of the detected alteration does not exclude a complex disease. Even rare and low-frequency variants have been shown to contribute to various complex diseases such as colorectal cancer [12]. The complex disease pathway of SSCD could for instance be identified by genetic association studies, but this would require a large pooling of patients.

The selection and number of patients represent a possible bias, which might explain the low number of detected sequence alterations. However, all patients were symptomatic, diagnosis was made based on HRCT with special reconstruction of the ideal plane to detect dehiscence, and seven patients were operated on and thus the dehiscence was confirmed. In case of a complex origin, the number of patients is not sufficient. Conversely, if the disease is monogenetic with multiple alterations like in α-thalassemia a careful selection of patients like in this study is of utmost importance and the detection rate is estimated to be high even in a low number of patients [13].

There is some evidence for a genetic etiology in SSCD. Further molecular-genetic research looking at the pathway of vestibular development must be performed before a definitive statement concerning a possible etiologic role for genetics in SSCD can be made.

## Conclusion

Histopathologic and radiologic studies point to a developmental origin of SSCD. Molecular analysis of two genes, which are known to be highly important for correct canal formation, has led to the detection of one sequence variant. Due to its low frequency and its conservative character the evidence for involvement in the formation of SSCD is low, in spite of its absence in the control population. However, a multifactorial origin cannot be excluded and the definite role of genetics in SSCD remains to be elucidated.

## Appendix

***Primers for genes HMX3 and Netrin-1***

Netrin2a-F 5′-TAATAACGACTCACTATAGGGAGCGCAGCTCCCTTCTCT-3′

Netrin2a-R 5′-ATTTAGGTGACACTATCAGAACTGCAGGCTCACGTA-3′

Netrin2b-F 5′-TAATACGACTCACTATAGGGAACGTCACGCTCACACTGTC-3′

Netrin2b-R 5′-ATTTAGGTGACACTATGACACCGCGTAGAAGTACGA-3′

Netrin2c-F 5′-TAATACGACTCACTATAGGGGGTCACGGCCACAGACAT-3′

Netrin2c-R 5′-ATTTAGGTGACACTATGAGCGAGTGGACCTGCTCT-3′

Netrin3-F 5′-TAATACGACTCACTATAGGGCGGCTGACACCTCTCTCTGT-3′

Netrin3-R 5′-ATTTAGGTGACACTATCTGATGTTCCCCGGGTCT-3′

Netrin4-F 5′-TAATACGACTCACTATAGGGCTTCCCCCTTGTCTGACC-3′

Netrin4-R 5′-ATTTAGGTGACACTATCCACAAAGCCAGAGACTTGA-3′

Netrin5-F 5′-TAATACGACTCACTATAGGGTCATGTCGTCTTACCTTGTTTTCT-3′

Netrin5-R 5′-ATTTAGGTGACACTATCAAAAGCGGGAAATGAGAAG-3′

Netrin6-F 5′-TAATACGACTCACTATAGGGCCTATTCATCGCCAGCCTAA-3′

Netrin6-R 5′-ATTTAGGTGACACTATAGGGTCTTCACGACCAACAC-3′

Netrin7.2-F1 5′-TAATACGACTCACTATAGGGGTGTATAAGCAGGGCACGAG-3′

Netrin7.2-F2 5′ATTTAGGTGACACTATGGACTGGTGGAAGTTCACG-3′

HMX3.1A-R1 5′TAATACGACTCACTATAGGGATCTCAAAGCGAGGGAAAGC-3′

HMX3.1A-R2 5′ATTTAGGTGACACTATGGGTCAGGGTGTAGGGGTA-3′

HMX3-2aF 5′TAATACGACTCACTATAGGGGCGTCCGTCTGTCTGTCTC-3′

HMX3-2aR 5′ATTTAGGTGACACTATCTGCCGCTTCCACTTGTT-3′

HMX3-2bF 5′TAATACGACTCACTATAGGGACCGAGACGCAGGTCAAG-3′

HMX3-2bR 5′ATTTAGGTGACACTATAAGGCCGCTGGACTTCTC-3′
